# Grounding abstract concepts and beliefs into experience: The embodied perspective

**DOI:** 10.3389/fpsyg.2022.943765

**Published:** 2022-07-22

**Authors:** Giovanni Buccino, Ivan Colagè

**Affiliations:** ^1^Divisione di Neuroscienze, IRCCS San Raffaele and Università Vita-Salute San Raffaele, Milan, Italy; ^2^Faculty of Philosophy, Pontifical University Antonianum, Rome, Italy; ^3^DISF Research Center, Pontifical University of the Holy Cross, Rome, Italy

**Keywords:** embodied language, abstract concepts, beliefs, semantic hub, Broca's region

## Introduction

The embodied perspective on language is now supported by several studies showing that activation of neural substrates processing the sensory and motor aspects of the world is not only associated with the processing of language referring to concrete aspects of the world (Buccino et al., 2005; Tettamanti et al., 2005; Gough et al., 2012, 2013; Marino et al., 2013, 2014; Visani et al., 2022) but is also *causal* to the understanding of concrete language (e.g., Bak et al., 2006; Sato et al., 2008; Kemmerer et al., 2012; Tremblay et al., 2012; Cardona et al., 2013; Fernandino et al., 2013; Desai et al., 2015; Klepp et al., 2015; Buccino et al., 2018; for review Buccino et al., 2016).

Less clear is the situation about abstract domains – i.e., language items referring to less concrete actions (e.g., “I give you my *opinion*”) or less tangible aspects of the world (e.g., “freedom”) (Glenberg et al., 2008; Boulenger et al., 2009). Such language items bring in a more conceptual and lexical-semantic dimension apparently less amenable to be understood in embodied terms. However, such abstract and conceptual domains are widespread in human linguistic practice and are, consequently, both problematic and interesting for the embodied approach (Gallese and Lakoff, 2005). Limited empirical findings and the variety of theoretical stances on abstract language (see Binder et al., 2009; Wang et al., 2010; Buccino et al., 2019 for reviews) have prompted “hybrid” models on abstract concepts and words. All these models share the embodied approach, but all also posit that acquisition and understanding of abstract concepts and words is only partially grounded in experience-related sensory-motor neural substrates and also resorts to supposed a-modal brain modules processing “pure” language aspects.

In the next section we will first briefly mention such hybrid models; then we will present a “fully embodied” approach (Buccino et al., 2019) and review the available evidence supporting it (Del Maschio et al., 2021). Finally, we will suggest how the advancements in embodied abstract language may shed light on the nature of beliefs.

## From embodied abstract language to beliefs

### The hybrid models for abstract language and concepts

Three main hybrid models for embodied abstract language have been proposed. The first model (Borghi et al., 2017, 2018) forwards that abstract words and concepts are mainly rooted in the social conventions and ensuing social interactions about abstract content, thus implying the existence of a brain system dedicated to processing propositional aspects of language. The second model maintains that specific features of words' meanings are indeed coded in sensory, motor or even emotional brain circuits. However, words' meaning is ultimately coded in specific, high-order, a-modal, linguistic regions, (Binder et al., 2009; Desai et al., 2015; Mahon, 2015) labeled as “semantic hubs”. The third model views the specificity of abstract words and concepts in the exalted emotional load they display and thus forwards that processing abstract contents specifically involves brain regions for coding, feeling and expressing emotions (Barsalou, 1999; Kousta et al., 2011; Moseley et al., 2012; Vigliocco et al., 2014). It is worth stressing here, however, that a number of studies (Wilson-Mendenhall et al., 2011, 2013) have shown that emotions themselves are grounded in the neural structures where the experiences and experiential contexts emotion word refer to are represented.

### A fully embodied approach

Buccino et al. (2019) forwarded a fully embodied approach to abstract language, that avoids assuming the existence of a-modal, purely linguistic systems to processing abstract words and concepts. This proposal is based on the idea that abstract words and concepts are such because of the complexity of the experiences attached to them, and not because they are far or detached from concrete experience. Specifically, such experiential complexity can increase according to (i) the number of effectors involved, (ii) the number of sensory systems engaged, as well as (iii) the accumulation over time of concrete life experiences (and related emotional load) attached to those words/concepts. Moreover, the distinction between abstract and concrete words/concepts may be one of degree and not of kind, as the complexity of experiences may increase along a continuum rather than sharply.

This approach allows for a strongly embodied interpretation of the evidence about the neural substrates processing abstract words, thus overcoming the need to elaborate hybrid models. Besides the data reviewed to advocate this fully embodied approach (see also Buccino et al., 2016, 2019), a recent meta-analysis of neuroimaging studies reporting activations related to abstract and concrete concepts further support this fully embodied approach (Del Maschio et al., 2021).

This meta-analysis shows that extensive clusters in the left temporal lobe (including the middle and inferior temporal gyri) and in the left motor cortex, as well as activations in right parietal cortex, left inferior frontal gyrus, and prefrontal regions are found for *both* concrete and abstract concepts. This suggests that (a) processing of these two kinds of concepts is not sharply segregated in the brain, (b) abstract concepts, like concrete ones, engage brain circuits involved in subjects' interaction with the world, and (c) abstract concepts are not pre-eminently processed in linguistic/propositional format, in semantic hubs or in emotion-related areas (in contrast to what hybrid models propose). Consequently, since semantic hubs are neural structures engaged by both concrete and abstract concepts, it is hard to accept the notion that they may be the “apex” of hierarchical structures progressively moving from processing concrete to abstract situations. Rather, these semantic hubs may play the role to contextualize actions (and related linguistic material) independently of their degree of abstractness.

The metanalysis by Del Maschio et al. (2021) also unveils that brain regions more active for abstract than concrete concepts encompass two major clusters in the left inferior frontal gyrus (pars triangularis and orbitalis, largely overlapping Broca's region) and middle temporal gyrus, as well as smaller clusters in medial frontal cortex and bilateral temporal poles.

According to the hybrid models, the stronger activation of Broca's region during the processing of abstract language supports the notion that abstract language is coded in a propositional format, since Broca's region is classically considered a linguistic region. In contrast with the classical view, many functions are now attributed to the Broca's region (Amunts and Zilles, 2012; Hardwick et al., 2018). First, in Broca's region there is a motor representation of mouth, hand-arm and, likely, foot actions (Binkofski et al., 1999; Nishitani et al., 2005). Secondly, Broca's region also processes observed and imagined actions (Binder et al., 2009; Hardwick et al., 2018). Thirdly, and more generally, there is also representation of mimicked actions, i.e., actions where the effector is used independently of the object (Lui et al., 2008); mimicked actions may be regarded as a first step in generalizing over object-oriented actions. Finally, Broca's region also codes actions able to mediate a semantic meaning, such as in emblems, but always using a biological effector (Andric et al., 2013).

All this suggests that Broca's region can support a process of generalization (indeed, of abstraction) of actions, but always starting from concrete situations and contexts: it might be said that Broca's region can grasp “what is common” to various instantiations of actions in varying contexts and situations. This view of Broca's activation is consistent with the notion that abstract language engages multiple effectors and contexts in which the use of the effector is not bound to specific objects. Put differently, because abstract concepts and their corresponding verbal labels express actions or entities that are dynamic in time and space, executed by different effectors, and coded in different systems (Buccino et al., 2019), their content, more strongly than for concrete concepts and words, is coded “motorically” in a brain region where actions are represented in a conceptual manner.

Other areas found more active for abstract language (specifically, medial frontal cortex and middle temporal gyrus) are indeed part of the proposed a-modal semantic hubs (Binder et al., 2009; Desai et al., 2015; Mahon, 2015). However, these brain regions are also known to be part of the “default-mode” network that is modulated by demanding cognitive tasks or by social cognition (Mars et al., 2012; Raichle, 2015); their engagement in processing abstract language (i.e., language items attached to complex experience) can be explained assuming that they may contribute to define an appropriate context for the processed words and their link with life experiences and personal beliefs.

Summing up, a fully embodied approach would account for the available data about processing of abstract language in the brain consistently with current knowledge of the functions of brain regions not directly involved in sensory-motor processing and without postulating the existence of a-modal, purely linguistic brain modules.

### Implications for belief

Beliefs are high mental processes implying abstract conceptualization and generalization. In this context, the notion of “belief” should be understood broadly, so to encompass moral contexts related to value and religion as well as cognitive convictions on how the world is done and works (and on how we should consequently behave in it). Moreover, beliefs should be conceptualized in strict connection with actions and life conduct. Philosopher C. Peirce stated that “a conception, that is, the rational purport of a word or other expression lies exclusively in its conceivable bearing upon the conduct of life” (Peirce, 1905; p. 162). A recent neuroscientific approach to beliefs indeed posits that selection of beliefs is virtually equivalent to selection of actions (Sugiura et al., 2015). The link between beliefs and actions, as well as the understanding of beliefs as conceptual items, suggests the relevance of an embodied approach to abstractness for the issue of beliefs.

Interestingly, recent developments in the neuroscience of action has established a link between action-related brain processes and the issue of beliefs and personal identity (Jeannerod, 2001, 2006, 2009; see also Colagè and Gobbi, 2017). According to this theory, the assessment of our experiences, especially the results of complex actions, may lead us to build up, and possibly revise, our belief system, which in turn allows for the planning of complex actions (Jeannerod, 2009, p. 263–269).

For this reason, a fully embodied approach to abstract words and concepts may shed light onto the process of building up and revising beliefs, specifically suggesting that beliefs, much like other conceptual domains, can be grounded in actual experiences and their complexity. Three further hints can be added.

First, we have seen that mesial pre-frontal cortex activates in processing abstract language and that this activation can be explained by the need to contextualize and frame abstract words on the background of one's life experiences. Specifically, studies suggest that this brain region is modulated, during the judgment of different relevant social situations and contexts, by the degree of similarity with our own beliefs, attitudes and inclinations (Mitchell et al., 2006; Zaki et al., 2014). Given the complexity of experiences attached to abstract words, these activations may help focusing on a relevant subset of the complex array of experiences attached to an abstract word. It is interesting to note that mesial pre-frontal cortex is proposed as key structure for processing beliefs, and specifically for integrating perception-, action- and emotion/value-related information (Seitz and Angel, 2012; Sugiura et al., 2015).

Secondly, mesial pre-frontal cortices are also known to be part of the mentalizing and affect-related brain systems (Frith and Frith, 2012). Activation of mesial frontal cortex in processing abstract words/concepts may reflect the need of the subject to retrieve his/her social and self-related beliefs to understand abstract linguistic items properly (see also Buccino and Colagè, 2017).

Finally, a fully embodied approach to beliefs is also consistent with the idea that such linguistic transactions among human beings are anyway grounded in real experiences. Linguistic transactions are effective in belief formation to the extent to which they help us sharing and combining our experiential baggage (Colagè and Buccino, 2016).

## Author contributions

GB conceptualized the paper and revised the manuscript. IC wrote the first draft of the manuscript and revised it. All authors contributed to the article and approved the submitted version.

## Funding

This paper is funded by Dr. Rüdiger Seitz, *via* the Volkswagen Foundation, Siemens Healthineers, and the Betz Foundation.

## Conflict of interest

The authors declare that the research was conducted in the absence of any commercial or financial relationships that could be construed as a potential conflict of interest.

## Publisher's note

All claims expressed in this article are solely those of the authors and do not necessarily represent those of their affiliated organizations, or those of the publisher, the editors and the reviewers. Any product that may be evaluated in this article, or claim that may be made by its manufacturer, is not guaranteed or endorsed by the publisher.
